# Mycoplasma, antibiotics in lay, and antimicrobial resistance (AMR)

**DOI:** 10.1016/j.psj.2026.107095

**Published:** 2026-05-07

**Authors:** C.J. Morrow, R.A. Achari, M. Charles, G.F. Browning, J.H.P. Ho, Cai Manshan, D.M. Bulach, Z. Kreizinger, M. Gyuranecz

**Affiliations:** a Bioproperties Pty. Ltd., Ringwood, Victoria, Australia; b Asia-Pacific Centre for Animal Health, Melbourne Veterinary School, The University of Melbourne, Victoria, Australia; c Innovative Diagnostics, Montpellier, France; d Agriculture, Fisheries and Conservation Department, Hong Kong Special Administrative Region of China; e State Key Laboratory of Swine and Poultry Breeding Industry, Guangdong Key Laboratory of Animal Breeding and Nutrition, Institute of Animal Science, Guangdong Academy of Agricultural Sciences, Guangzhou 510640, China; f MolliScience, HUN-REN Veterinary Medical Research Institute, Budapest, Hungary

**Keywords:** Antimicrobial resistance, *Mycoplasma synoviae*, *Mycoplasma gallisepticum*, Mycoplasma freedom, Live mycoplasma vaccination

## Abstract

Antimicrobial resistance (AMR) is an emerging problem in human health from the way that we have used antimicrobials. To keep the benefits of antimicrobials there is a need to change the way they have been used. Preventive treatments need to be phased out especially in intensive animal production. In poultry meat and egg production antimicrobials have often been used regularly in the laying period. Evidence presented at this symposium that this is driving antimicrobial resistance in *Mycoplasma gallisepticum* and *M. synoviae* where prevention allows benefits to accrue but also resistance in chicken microbiotia including zoonoses to develop. Mycoplasma freedom and/or live mycoplasma vaccination can decrease antibiotic dependence and must be the way ahead.

## Introduction

At first glance the Mycoplasma, antibiotics in lay and Anti-Microbial Resistance (AMR) symposium (14 October 2025) held in Macau as part of the Pacific Rim Scientific Conference of the Poultry Science Association, seems to be a conglomeration of vaguely related papers with little overlap, but participants argued that antimicrobial resistance in poultry poses a risk to human health and has its genesis in the use of antimicrobials in chicken meat and egg production. It was suggested that a major reason for the use of antimicrobials in laying birds is to control the effects of infection with *Mycoplasma gallisepticum* (MG) and *M. synoviae* (MS) in lay (Morrow, 2024). The role of the emergence of resistance, and especially multiple resistance (Morrow et al., 2020), in MG and MS in driving the use of ever-increasing numbers of antimicrobials (from our finite range of potential options) was also discussed. The information presented at the symposium attempted to look at these major uses of antimicrobials in terms of the consequences of the magnitude of the problem of antimicrobial resistance, the dynamics of this evolving problem and the downstream effects of routine antimicrobial use, including driving antimicrobial resistance in the microbiota of chickens.

By the 1990s antibiotics were effectively marketed into chicken production systems and used in lay to control the effects of avian mycoplasma infections (Kleven, 2008). In Asia and other high use regions, fluoroquinolone resistance rapidly emerged in avian mycoplasmas. The problem has been exacerbated by routine use of antimicrobials to control post-vaccinal reactions commonly associated with LaSota NDV vaccination of mycoplasma positive broilers. In addition, antimicrobials have also commonly been added to diets as “growth promoters”. This use of antimicrobials was also commonly thought to control clostridial infections, but inclusion of tylosin would also reduce the impact of infection with MG. Although this practice seems to have been largely phased out at feed mills, some work-arounds, including adding antimicrobials to feed at the farm or during delivery of the feed, have been introduced to circumvent the ban on addition of antimicrobials to feed at the mill.

Over time antimicrobial administration in lay became increasingly adopted, with companies competing to sign up integrations and layer farmers as competitor products failed. To farmers it seemed that antimicrobials were an essential supplement required in poultry feed. However, the chronic nature of MG and MS infections in chickens explains why production benefits are seen in infected flocks. Thus, the effect is not a result of a general effect of antimicrobials on bacteria, but rather a consequence of continual suppression of MG and MS, decreasing the biological cost of the response of chickens to these infections.

Mycoplasmas are not sensitive to all antimicrobials. They have no cell walls and are inherently resistant to those antimicrobials that interfere with cell wall synthesis, including penicillins, colistin and cephalosporins. They are also innately resistant to sulphonamides as a consequence of their highly host adapted metabolism and parasitic lifestyle. MS is also innately resistant to erythromycin.

The treatment of clinical respiratory disease in broilers is sometimes investigated by assessment of the antimicrobial resistance of *E. coli,* but the importance of mycoplasmas or viruses is not assessed in many Asian countries.

Resistance in mycoplasmas to single antimicrobial drugs was a significant problem in the 1980s (Gautier-Bouchardon, 2018). Initially this was easily handled by changing antimicrobial being used. In Asia, laboratory investigation of resistance was limited to some big integrations with in-house laboratories. In part, investigations were limited by the difficulties of isolating mycoplasmas in tropical laboratories.

## Discussion

Prof. Browning described the role and importance of antimicrobial stewardship in combatting antimicrobial resistance and warned that unnecessary use selects for resistance in target and off-target bacteria. Prophylactic or metaphylactic use of antimicrobials is often empirical and done without diagnostic support. It is hard to tell if use is beneficial without knowing if the flock is undergoing challenge. A focus on governance does not provide solutions to all the underlying drivers of use. Vaccines are important alternatives to use of antimicrobials, especially if they also decrease transmission. A lack of access to sophisticated diagnostic laboratories may see clinicians concentrating on cultivable bacteria, which may be secondary to primary infection with mycoplasmas and/or viruses. The reporting of antimicrobial resistance in commensal *E. coli* in the microbiota of chickens in some Asian countries has sometimes been mistaken locally as a guide to therapy of clinical cases, with a focus on selecting drugs to which very few or no organisms are resistant, rather than on ensuring that antimicrobials are used selectively, and only when absolutely necessary, and that choices for therapy need to be restricted to drugs that have low importance in human medicine to control selection for resistance. Combination products are commonly used in some countries, with up to 5 different antimicrobial drugs including in some products in a misguided attempt to achieve the broadest spectrum possible for empirical treatment. Education and diagnostic support are critical needs to ensure better outcomes and reduce inappropriate use of antimicrobials. Mycoplasma vaccination is likely to be an important intervention to decrease antimicrobial use across the region, although use of the F strain *M. gallisepticum* in breeder flocks may be a contributor to ongoing endemicity of MG in broilers that is driving use of antimicrobials to treat secondary colibacillosis.

Dr Ho reported that the Government of the Hong Kong Special Administrative Region (HKSAR) has launched the Hong Kong Strategy and Action Plan on Antimicrobial Resistance (AMR) since July 2017 (The Government of HKSAR, 2017). Under this Action Plan, the Agriculture, Fisheries and Conservation Department (AFCD) has established a comprehensive surveillance system monitoring antimicrobial usage (AMU) and AMR in local poultry farms since 2019. Based on the surveillance outcomes in local poultry farms, low stable levels of extended-spectrum beta-lactamase-producing *E. coli* (ESBL-E) and carbapenem-resistant *E. coli* (CRE) persist over the years despite the extremely low AMU, suggesting the lack of correlation between AMU and AMR in local poultry. Indeed, no vancomycin-resistant enterococci (VRE) have been detected in the monitoring to date. Additional studies indicated that local poultry farm environments may serve as reservoirs for ESBL-E, prompting targeted interventions such as educational campaigns on feed handling, cleaning and disinfection, and the exploration of competitive exclusion via probiotics. Further interventions include the “veterinary-prescription only medication supply” policy, which started in 2025 in Hong Kong requiring mandatory veterinary prescription for purchase and use of antimicrobials on farm animals (The Government of HKSAR, 2022). The AFCD’s integrated strategy—encompassing surveillance, prudent antimicrobial stewardship, enhanced biosecurity, and sustained education and training— is a model for similar AMR surveillance and control programmes in the region.

Over the last decade, avian mycoplasmas have been isolated by filtering samples at the time of collection. This has allowed isolates to be made of South-East (SE) Asian strains of MG and MS. In medical diagnostic laboratories, three isolates are usually characterized from one sample, whereas in poultry sampling swabs from multiple birds are pooled, and the organisms in a sample with the highest *in vitro* fitness will be the ones isolated.

MIC determination is mostly done using a broth microdilution method. Unfortunately, assessment of antimicrobial resistance in avian mycoplasmas is not yet standardized. The lack of standard control strains, a universal medium and standardised interpretation guidelines are some of the confounding problems. Dr Kreizinger noted that measuring the initial MIC (iMIC) is the appropriate technique to use for antimicrobials that are bacteriostatic (nearly all anti-mycoplasmal antibiotics, except fluoroquinolones). This technique requires that the plate be read when the control (no antibiotic) wells first change colour. Recording after a fixed time defines the final MIC (fMIC), which is only appropriate for bactericidal antibiotics. It is rare to obtain a result from sampling in less than one month, so susceptibility testing is of little use for determining the appropriate treatment of the flock that was sampled (Gautier-Bouchardon, 2018).

The use of susceptibility testing employing microtitre plates containing pre-diluted antimicrobials, rather than just testing clinical resistance to a therapeutic intervention, helped identify multi-resistant MG and MS strains. Resistance or partial resistance has been detected to all the antimicrobials tested, including fluroquinolones, tetracyclines, macrolides (spiramycin, erythromycin, tiamulin, tylosin, tilmicosin and tylvalosin, lincomycin, florfenicol and the aminocyclitol spectinomycin) in isolates from the region.

Prof Gyuranecz described known antimicrobial resistance mechanisms in avian mycoplasmas, observing that the initial response of most mycoplasmas is increasing efflux, but this often produces limited antimicrobial focused resistance and is probably the explanation of dosage creepage regularly seen in the field after sourcing of a new active antimicrobial. More dramatic resistance to multiple antimicrobials can be seen with modified efflux mechanisms. Mutations in the gene encoding the target of antimicrobial can result in strong resistance and has been described for fluoroquinolones and some macrolides (and lincomycin), but it is suspected that more of this resistance will be detected with further research. Biofilms are also emerging as an important mechanism contributing to multi-drug resistance. Drug inactivation does not seem to have been seen as a resistance mechanism in avian mycoplasmas to date.

Some research has been done to design PCR-based tests that can detect antimicrobial resistance in MG and MS. This has concentrated on changes in target genes associated with the acquisition of resistance, but these tests have not yet been particularly useful.

Dr Bulach reported on whole genome sequencing of a selection of novel multi-drug-resistant MG and MS isolates from SE Asia. Although some multi-resistant strains have target gene mutations associated with antimicrobial resistance, not all the multi-resistant isolates had these changes (Lysnyansky and others, 2012). One multi-resistant Vietnamese MG isolate had an allele like a MG gene reported to be associated with biofilm elaboration and multiple antibiotic resistance (Ma and others, 2023).

The implications of this emergence of multi-resistance are concerning from both a veterinary clinical perspective (because of the potential for vertical transmission of organisms that cannot be treated effectively with any antimicrobials) and a public health perspective (because of the increasingly restricted range of effective treatments for recognised zoonotic pathogens – primarily salmonellas and campylobacters).

Dr Charles reported on a new large survey (97 participants) of mycoplasma control strategies in SE Asian (14 countries) poultry integrations. The customers surveyed were mainly acquiring mycoplasma-free replacement stock, but sometimes they were being cross-bred with local breeds, which may have had MG and MS infections, or were being introduced onto multi-age sites. MG control and MS control were seen by many respondents as different or independent. Approximately 30% of respondents reported regularly using routine antimicrobial treatment for mycoplasma control. Live and killed vaccines for MG were used by 35% of respondents and live MS vaccination by 41% of them.

Dr Cai Manshan reported on success in mycoplasma control in yellow chickens in southern China. In 2015, MS was causing infectious synovitis in 27% of broilers after a 6% cull. Antimicrobials had variable efficacy, so the MS-H vaccine was tried. Laboratory support, including DIVA PCR, aided decision making and assessment of the success of the use of live mycoplasma vaccines. Two applications of the vaccine and treatment with flushing antimicrobials before the first vaccination, with no subsequent antimicrobial treatment in lay was the best control in terms of preventing colonisation with wild type MS.

This report of success was augmented by Dr Achari’s report of mycoplasma control using the live vaccines MS-H and ts-11 in Asia and the rest of the world. This is sometimes done in the face of vertical infection of replacement stock. Further education is needed in this area to encourage wider adoption of the ability to supply vertically uncontaminated stock, improved biosecurity, the elimination of antibiotics to prevent “post vaccinal reactions” in broiler progeny of mycoplasma protected vaccinated parent stock and the complete phasing out of the treatments of clinically healthy flocks (removal of routine antibiotics). There are differences between the current live vaccines on the market in terms of the convenience of use, their capacity to transmit vertically, the availability of DIVA assays to distinguish vaccinated flocks from those experiencing wild type challenge, and reports about their performance. They are all largely sensitive to anti-mycoplasmal antibiotics.

Dr Morrow report noted that in China, Vietnam and Taiwan, yellow broiler chicken breeder operations suffer from an inability to supply MS-free broiler chickens, driving a lot of antimicrobial use, and the emergence of multi-resistance is probably a consequence of poor governance of the use of antimicrobials. Biosecurity is often poor in the traditional farming systems and multi-age breeder sites are commonly used throughout the breeding pyramid. Experience in Europe has shown that MS can be transmitted horizontally by aerosols over a distance of 2 km. Sheds 10 m apart will result in early challenge of replacement stock with endemic mycoplasmas.

Layer stock have had a problem with Egg Apical Abnormalities caused by MS infection in SE Asia. The 2019 ban on the addition of antimicrobials to feed in China is resulting in massive reductions in antimicrobial use in poultry, including in layers.

## Conclusion

This symposium described the major One Health problem caused by the way antimicrobials are currently used in Asia and other regions in poultry meat and egg production. The problem is becoming more urgent, with multi-resistance increasing in MG and MS. The solution is to control mycoplasmas by establishing mycoplasma-free breeder flocks or by vaccination. Practical solutions were described, and strategic application of these solutions should minimise antimicrobial use and its consequent problems. Good biosecurity is still needed and should be built into new farms and integrations, but current levels of antimicrobial use can still be markedly decreased using currently available tools for control.

## CRediT authorship contribution statement

**C.J. Morrow:** Writing – review & editing, Writing – original draft, Investigation, Conceptualization. **R.A. Achari:** Investigation, Formal analysis. **M. Charles:** Investigation, Formal analysis. **G.F. Browning:** Writing – review & editing, Investigation, Conceptualization. **J.H.P. Ho:** Writing – review & editing, Investigation, Formal analysis. **Cai Manshan:** Writing – review & editing, Investigation, Formal analysis. **D.M. Bulach:** Writing – review & editing, Methodology, Investigation, Formal analysis. **Z. Kreizinger:** Writing – review & editing, Investigation, Formal analysis. **M. Gyuranecz:** Writing – review & editing, Investigation, Formal analysis.

## Disclosures

The authors declare no conflict of interest. The following is an explanation of their involvement in the subject of the symposium.

Chris Morrow has recently retired from the Global Technical manager position of Bioproperties and still works for this company as a consultant. He is also an Honorary Researcher at the University of Melbourne. He receives a royalty through the University of Melbourne for a vaccine (Mycoplasma synoviae) that he invented in 1988 and this vaccine was licensed to Bioproperties. This vaccine is the only live *Mycoplasma synoviae* vaccine available in the world at the moment. (MSD did have one called MS1 which was briefly on the market around the world but is no longer registered or used). 30 years ago he worked for Ross Breeders (Aviagen) servicing customers in the Asian region and other parts of the world for a decade.

Dr. Robin Achari is the new Global Technical manager of Bioproperties.

Magali Charles has previously worked for Cobb for 5 years in Asia but currently works for a French Diagnostic company.

Prof. Glenn Browning is a veterinary microbiologist who also has been involved in vaccine development at the University of Melbourne. He also has projects funded by the Fleming Fund on AMR in Asia. He is the director of APCAH (Asia-Pacific Centre for Animal Health). Dr Dieter Bulach is a microbiologist and bioinformatics expert currently working for a Fleming funded project led by Glenn Browning.

Dr. Cai Manshan is the veterinary manager of a yellow chicken integration in Southern China with a university position

Prof Miklos Gyuranecz and Dr Zsuzsa Kreizinger specialize in isolation and analysis of veterinary mycoplasmas based at the Veterinary Research institute in Budapest.

Dr Jeremy Ho works for the Hong Kong government.
